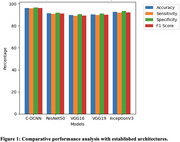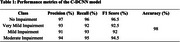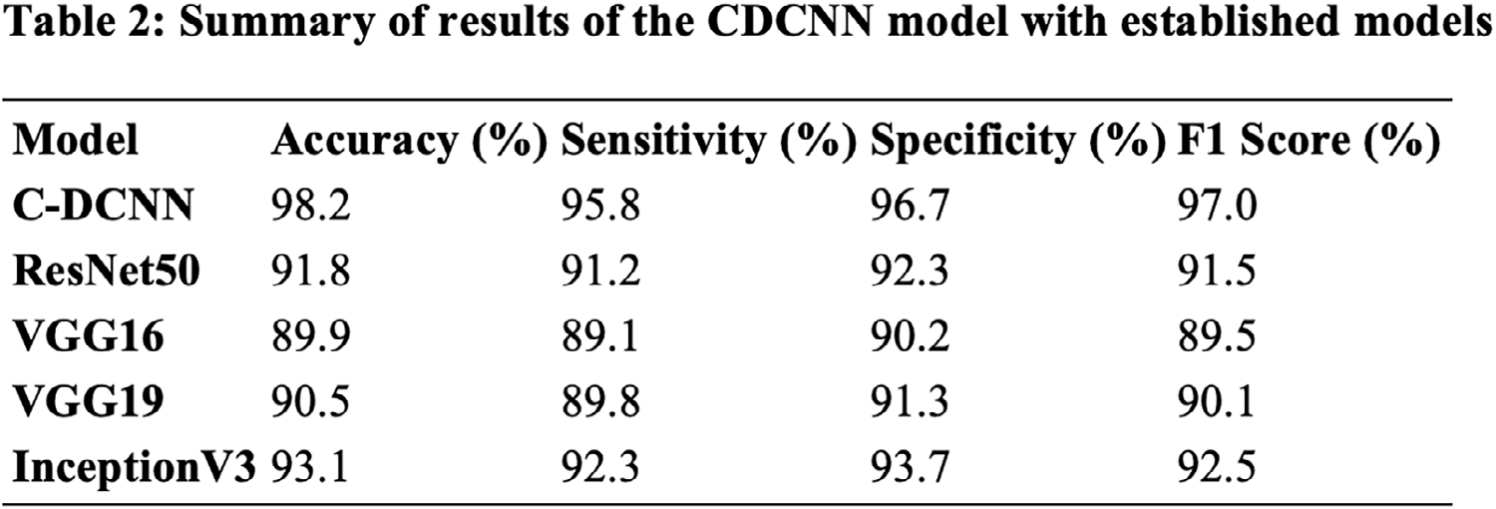# Enhancing dementia classification using denoising diffusion models and conditional deep convolutional neural networks

**DOI:** 10.1002/alz70856_098727

**Published:** 2025-12-24

**Authors:** Efe Precious Onakpojeruo, Dilber Uzun Ozsahin, Berna Uzun, Ilker Ozsahin

**Affiliations:** ^1^ Operational Research Center in Healthcare, Near East University, Nicosia/TRNC, Mersin 10, Turkey; ^2^ Medical Diagnostic Imaging Department, College of Health Sciences, University of Sharjah, 27272, Sharjah, United Arab Emirates

## Abstract

**Background:**

The imbalance in medical imaging datasets and concerns over patient privacy present challenges in AI‐driven dementia diagnosis. This study leverages synthetic data generated by Denoising Diffusion Models (DDM) to address these issues, marking a significant advancement over traditional GAN‐based augmentation techniques.

**Method:**

Using the Kaggle Alzheimer's MRI dataset as a base, this research pioneers the integration of DDM with a novel Conditional Deep Convolutional Neural Network (C‐DCNN) for classifying dementia stages. The dataset comprises axial MRIs of four categories: No Impairment, Very Mild Impairment, Mild Impairment, and Moderate Impairment, balanced with 2,560 synthetic images per class. Preprocessing included skull removal, grayscale conversion, and resizing to 128×128 pixels. The DDM‐generated synthetic images were validated by a radiologist to ensure clinical relevance.

**Result:**

The proposed C‐DCNN model achieved a remarkable accuracy of 98%, significantly outperforming established architectures like ResNet50, VGG16, VGG19, and InceptionV3.

**Conclusion:**

This study demonstrates the efficacy of DDM‐generated synthetic datasets in improving dementia classification accuracy, setting a new benchmark for AI applications in healthcare. The proposed framework offers a scalable and robust solution for early diagnosis and treatment planning in dementia care.